# Supplementary material for: Structural and Functional Validation of a Full-Thickness Self-Assembled Skin Equivalent for Disease Modeling
Source: Pharmaceutics. 2022 Jun 7;14(6):1211. doi: 10.3390/pharmaceutics14061211 (PMC9231172; doi:10.3390/pharmaceutics14061211)
Supplement: Supplementary file 1 [file pharmaceutics-14-01211-s001.zip › pharmaceutics-1740802-supplementary.pdf]

pharmaceutics

Supplementary Materials: Structural and functional validation of a full-thickness self-assembled skin equivalent for disease modeling

Bo Ram Mok, Su Ji Shon, A Ram Kim, Dong Hyun Kim, Jung U Shin

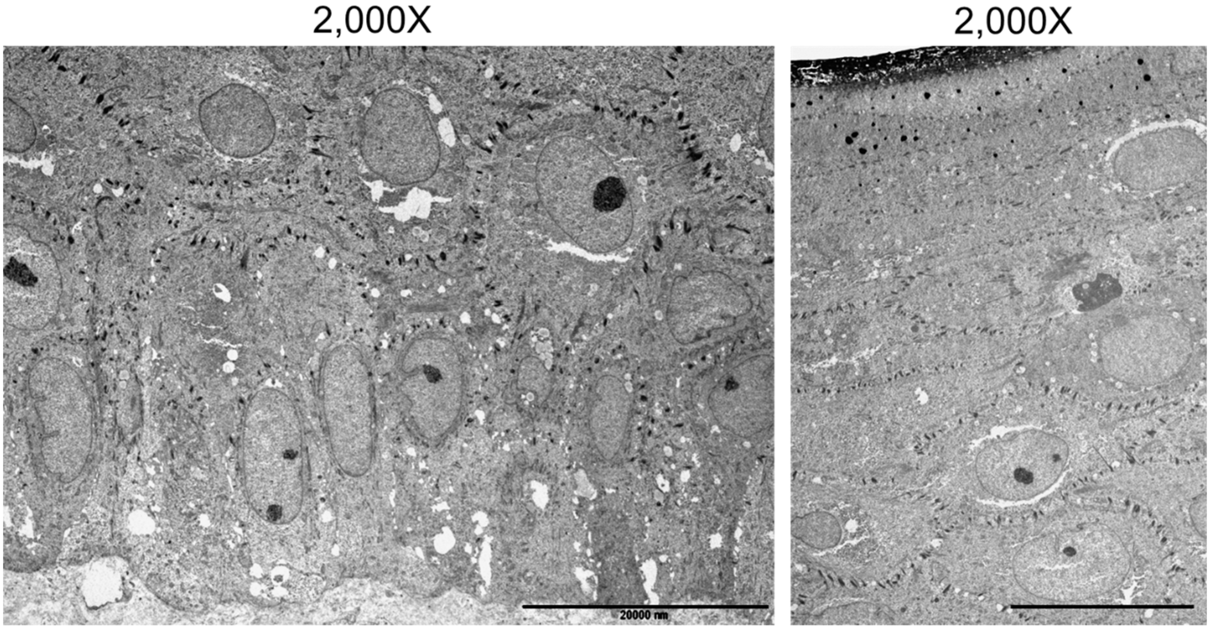

Figure S1. The structure of the epidermal layer observed by TEM. The entire epidermal structure was observed with a transmission electron microscope. Columnar keratinocytes were found in the basal layer, and the shape of the cells became flattered as they went up, and the nucleus disappeared as they approached the stratum corneum. Also, it can be seen that desmosomes are closely arranged between cells.

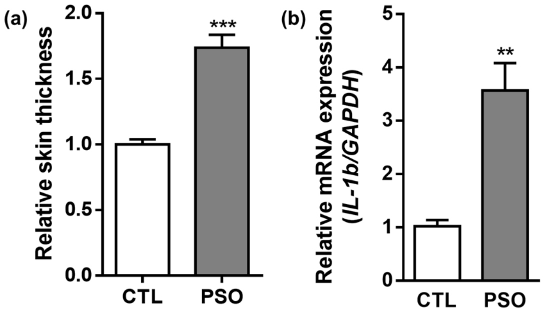

Figure S2. Comparison of control RSE and psoriatic RSE. (**a**) Skin thickness of control and psoriatic RSE. The psoriatic RSE model shows a thicker epidermis than the control RSE. (**b**) The *IL-1b* mRNA expression in control- and PSO-RSE. The increased expression of *IL-1b* mRNA is observed in the self-assembled psoriatic RSE models. RSE: Reconstructed skin equivalent; PSO: Psoriatic RSE. ** $p < 0.01$; *** $p < 0.005$.
